# Effect of short-term extremely low-frequency electromagnetic field on respiratory functions

**DOI:** 10.1590/1806-9282.20241812

**Published:** 2025-06-02

**Authors:** Ferhat Sirinyildiz, Gökhan Cesur, Onur Elmas, Sinem Elmas, Selcuk Comlekci, Onur Yazıcı, Adem Keskin, Huseyin Eren Keskin

**Affiliations:** 1 Aydin Adnan Menderes University, Faculty of Medicine, Department of Physiology – Aydın, Turkey.; 2 Süleyman Demirel University, Faculty of Medicine, Department of Physiology – Isparta, Turkey.; 3 Mugla Sitki Kocman University, Faculty of Sciences, Department of Biology – Muğla, Turkey.; 4 Süleyman Demirel University, Faculty of Engineering and Architecture, Department of Electronics and Communication Engineering – Isparta, Turkey.; 5 Aydin Adnan Menderes University, Faculty of Medicine, Department of Chest Diseases – Aydın, Turkey.; 6 Aydin Adnan Menderes University, Faculty of Medicine, Department of Medical Biochemistry – Aydın, Turkey.; 7 Yıldız Technical University, Faculty of Mechanical Engineering, Department of Mechatronics Engineering – İstanbul, Turkey.

**Keywords:** Electromagnetic field, Respiratory rate, Inspiration, Respiratory, Tidal volume

## Abstract

**OBJECTIVE:**

Exposure to extremely low-frequency electromagnetic fields can cause harmful or beneficial effects on living organisms. The aim of this study was to examine the potential effects of extremely low-frequency electromagnetic fields on respiratory physiology by investigating possible changes in respiratory function parameters during and after short-term extremely low-frequency electromagnetic field exposure.

**METHODS:**

Twenty Wistar albino rats were included in the study, and these rats were randomly divided into two groups: control and electromagnetic field. A noninvasive head-out plethysmography technique was used to accurately assess lung function in rats. Rats in the electromagnetic field group were exposed to electromagnetic field at a frequency of 50 Hz and a magnetic flux density of 0.3 mT for 2 min. Respiratory function parameters of both groups were recorded in three separate periods before, during and after electromagnetic field exposure. Respiratory rate, respiratory cycle duration, inspiration time, expiration time, tidal volume, minute volume, peak inspiratory flow, and peak expiratory flow were measured in these periods.

**RESULTS:**

There was no significant difference in the parameters measured before electromagnetic field exposure between the groups. During the electromagnetic field exposure period, the mean respiratory rate measured in the electromagnetic field group was lower compared to the control group data, while the mean respiratory cycle duration, inspiration time, and tidal volume measured in the electromagnetic field group were higher compared to the control group data. There was no significant difference in the parameters measured after electromagnetic field exposure between the groups.

**CONCLUSION:**

Short-term extremely low-frequency electromagnetic field exposure decreases respiratory rate and increases respiratory cycle duration, inspiration time, and tidal volume.

## INTRODUCTION

The development of new industrial activities and technologies is increasing both the number and diversity of electromagnetic field (EMF) sources globally. The main sources of EMF include mobile phones, audio and video equipment, various household appliances, radio stations, high-voltage power lines, and radar systems. With the widespread use of electronic devices, the effects of EMF on human health are of increasing interest to scientists^1^. EMFs, especially those produced by electrical devices and power systems operating in the range of 1 to 300 Hz, are defined as extremely low-frequency electromagnetic fields (ELF-EMFs)^2^. It is suggested that the effectiveness of EMFs, which can affect biological processes, is due to their fundamental effects on the plasma membrane due to their electrical properties^3^. On the other hand, it is reported that the effect of ELF-EMFs depends on a certain stage of the cell cycle or a certain metabolic state of the cells^1^.

EMF, which affects many biological processes, has attracted great interest due to its potential therapeutic and diagnostic implications in many diseases, such as musculoskeletal disorders and neurodegenerative diseases. In addition, EMF therapy is considered a promising noninvasive and nonionizing approach to treat cardiovascular diseases. However, although evidence has accumulated showing beneficial effects of EMFs in multiple cardiovascular diseases, the parameters used in these studies are not consistent. Therefore, basic physiological studies must be conducted to reveal the precise mechanism by which the cardiovascular system perceives external EMFs^4^. On the other hand, some original studies reported damaging effects on DNA molecules in cells exposed to EMFs, while other studies suggested that no such damage occurred in cells exposed to EMFs. These conflicting findings may be due to differences in the apparatus used to generate electromagnetic fields, experimental design, duration of exposure, genetic endpoints, and biological materials such as cell lines and animal species, strains, and age^5^. Additionally, long-term electromagnetic stress alters mitochondrial respiratory capacity. Furthermore, long-term exposure to EMFs may affect mitochondrial oxidative metabolism by affecting the cardiac oxidative phosphorylation system^6^.

ELF-EMFs have beneficial or detrimental effects on living organisms by regulating the oxidative–antioxidative balance in cells. Depending on the exposure time, ELF-EMFs can affect cell cycle regulation of the human lung fibroblast cell line Medical Research Council cell strain-5 through modulation of the oxidative/antioxidative defense system^7^. Experimental and clinical studies have shown that EMFs can have profound effects on peripheral nerves. Moreover, the effects of EMFs on the peripheral nervous system are more complex^8^. On the other hand, numerous studies have shown that EMF weakens body systems and leads to increased infection rates, including bacterial and viral infections. While Fifth-Generation (5G) Wireless Telecommunications is not a cause of coronavirus-19 infection, 5G exposure has been shown to be a statistically significant factor associated with higher coronavirus-19 cases and deaths in the United States^9^.

The widespread use of electronic devices and telecommunication technologies increases the diversity of EMF sources and creates the need to evaluate the potential effects of these fields on human health. The aim of this study was to examine the potential effects of ELF-EMFs on respiratory physiology by investigating possible changes in pulmonary function parameters during and after exposure to ELF-EMFs.

## METHODS

Before starting the study, the necessary ethics committee decision was obtained from the Laboratory Animals Local Ethics Committee of Süleyman Demirel University. A total of 20 female Wistar albino rats weighing approximately 185–215 g were included in the study, and these rats were randomly divided into two groups as control (not exposed to EMF, n: 10) and EMFs (exposed to EMF, n: 10). Rats were anesthetized by intraperitoneal administration of 50 mg/kg ketamine (Ketasol, Richter Pharma, Australia) and 10 mg/kg xylazine (Rompun, Bayer, Leverkusen, Germany). After anesthesia, rats were taken to the respiratory chamber and placed between Helmholtz coils. Their necks were placed outside the respiratory chamber with a collar made of dental latex. A noninvasive head-out plethysmography technique was used to accurately assess lung function in rats^10^.

In the experiment, Helmholtz coils [Coil wire: Copper, 22 AWG (0.64 mm diameter)] were fed in series. The Helmholtz coil assembly consists of two 500-winding coils (inner diameter 18.6 cm and outer diameter 22.1 cm) placed parallel to each other at 2.35 cm. When the wave generator was set to 5V DC, 120-mA current passed through the coils. As a result of the calculations, a magnetic field strength of 0.3 mT was created between the coils with this current and the maximum usable output voltage peak value of 10 V was obtained when the device output was regulated with a 120-mA square wave as the root mean square value.

Recordings were taken with an 8/35 data logger, a dual Amp bio-amplifier mounted on the device, an FE141 spirometer, and an ML325 animal pulse oximeter pod with an ML325/AW wrap sensor (Ad instruments, New South Wales, Australia). 50 Hz filtering and 1 k/s sampling rate were selected in the instrument software (Lab Chart 8 Pro, Ad instruments, New South Wales, Australia).

Anesthesia may cause changes in some physiological parameters such as heart rate and electroencephalogram^11,12^. Therefore, rats in the EMF group were started to be exposed to EMFs at a frequency of 50 Hz and a magnetic flux density of 0.3 mT at the 20th minute after anesthesia and terminated at the 22nd minute. Respiratory function parameters of the rats in the EMF group were recorded in three separate periods before (18–20 min), during (20–22 min), and after (22–24 min) EMF exposure. Respiratory rate, respiratory cycle duration, inspiration time, expiration time, tidal volume, minute volume, peak inspiratory flow, and peak expiratory flow were measured during these periods. In the control group rats, the same parameters were recorded in the same periods without EMF administration.

Statistical Package for the Social Sciences for Windows 22.0 (IBM, NY, USA) program was used for statistical analysis in evaluating the data in the study. Continuous data were expressed as mean±standard deviation. The Mann-Whitney U test was used to compare data between groups. p-values less than 0.05 were considered statistically significant.

## RESULTS

Respiratory function parameters of the groups before, during, and after exposure in three different periods are presented in Table 1.

**Table 1:** Respiratory function parameters of the groups before, during, and after exposure in three different periods.

Parameters	Groups	Pre-EMF exposure period (18–20 min)	EMF exposure period (20–22 min)	Post-EMF exposure period (22–24 min)
Respiratory rate (l/min)	EMF	56.52±5.72	52.41±5.13	55.93±5.91
	Control	57.21±5.59	57.43±5.45	58.08±5.64
Respiratory cycle duration (s)	EMF	1.06±0.12	1.15±0.13	1.08±0.14
	Control	1.05±0.12	1.04±0.12	1.04±0.13
Inspiration time (s)	EMF	0.36±0.05	0.40±0.05	0.35±0.05
	Control	0.36±0.05	0.35±0.05	0.34±0.05
Expiration time (s)	EMF	0.70±0.08	0.75±0.09	0.72±0.09
	Control	0.69±0.08	0.70±0.08	0.70±0.08
Tidal volume (mL)	EMF	1.53±0.17	1.69±0.18	1.51±0.19
	Control	1.54±0.19	1.53±0.18	1.49±0.17
Minute volume (mL)	EMF	86.42±14.33	88.50±14.14	83.83±14.52
	Control	88.11±14.06	87.80±13.92	85.78±14.44
Peak inspiratory flow (mL/s)	EMF	5.81±1.36	5.67±1.35	5.30±1.42
	Control	5.45±1.41	5.58±1.46	5.69±1.53
Peak expiratory flow (mL/s)	EMF	3.17±0.61	3.48±0.62	3.33±0.64
	Control	3.11±0.60	3.24±0.62	3.32±0.63

EMF: electromagnetic field.

No significant difference was found in the parameters measured in the pre-EMF exposure period (18–20 min) between the groups (p>0.05) (Table 1).

During the EMF exposure period (20–22 min), the mean respiratory rate measured in the EMF group was found to be lower than the mean respiratory rate measured in the control group (p=0.03) (Table 1). Furthermore, during this period, the mean respiratory cycle duration, inspiration time, and tidal volume measured in the EMF group were found to be higher than the mean respiratory cycle duration, inspiration time, and tidal volume measured in the control group (p=0.04, p=0.02, p=0.04, respectively) (Table 1). No significant difference was found in other parameters measured during the EMF exposure period (20–22 min) between the groups (p>0.05) (Table 1).

No significant difference was found in the parameters measured in the post-EMF exposure period (22–24 min) between the groups (p>0.05) (Table 1).

## DISCUSSION

In urban public spaces, exposure to ELF-EMFs typically ranges from 0.05 to 0.2 μT, while indoors, near instruments held close to the body, exposure levels can be as high as millitesla (mT)^13^. Epidemiological studies to determine whether exposure to EMF is a potential health risk factor have shown conflicting results, but EMF is used for diagnostic and therapeutic purposes in various medical fields such as rheumatology, neurology, orthopedics, and psychiatry. Although the mechanisms by which EMFs operate are still being studied, some researchers propose that exposure to ELF-EMFs may influence cell function by exerting mechanical effects on proteins within the cell and on the cell membrane, including ion channels, membrane receptors, and enzymes. Research consistently indicates that the impact of sinusoidal ELF-EMFs differs depending on the cell type and factors such as frequency, flux density, and duration of exposure^14^.

Increased ELF-EMF exposure raises concerns about potential adverse health effects from ELF-EMF. The central nervous system is expected to be particularly vulnerable to ELF-EMF because its function is largely dependent on electrical excitability. On the other hand, the neurotoxic risk of ELF-EMF exposure appears to be limited. Additionally, it is noteworthy that both acute and subchronic 50 Hz ELF-EMF exposures up to 1,000 μT have not consistently shown effects on Ca^2+^ homeostasis, reactive oxygen species production, or membrane integrity^15^. On the other hand, long-term (chronic) exposure to ELF-EMFs affects immune responses by stimulating the production of proinflammatory cytokines such as interleukin-1β, interleukin-6, and tumor necrosis factor-α and by increasing hematological parameters such as white blood cell count, red blood cell count, lymphocyte percentage, mean corpuscular volume, platelet count, and procalcitonin^16^. Similarly, a study in which rats were exposed to ELF-EMFs with magnetic flux densities of 1, 100, 500, and 2,000 μT reported increased levels of proinflammatory cytokines such as interleukin-9 and TNF-α when exposed to ELF-EMFs with magnetic flux densities of 100, 500, and 2,000 μT^17^.

A recent study reported that cellular response is determined by the combination of different ELF-EMF exposure parameters. Furthermore, the intensity parameter has gained great importance when establishing regulations regarding exposure to low-frequency electromagnetic fields. Intensity is considered a determining parameter for the emergence of cellular effects. Furthermore, the "dose effect" theory is one of the main approaches explaining these effects. This has also led to the use of intensity as the main parameter in in vitro experiments^18^. On the other hand, the intensity of EMF exposure produces unpredictable effects as shown by nonlinear effects. This is most likely due to the ability of the biological system to adjust and compensate but may eventually lead to biochemical deterioration after prolonged exposure. A recent study examining 112 low-intensity studies reported that intensity and duration of exposure interact because the absorbed energy dose is the product of intensity and time, and as a result, EMFs act as a biological "stress factor" that can affect a wide variety of living systems. In addition, it is clear that the biological outcome of varying the intensity and duration of EMF exposure is fundamentally unpredictable. This is mainly due to the complex nature of the biological system being studied. Intensity and duration can interact with the biological system and produce different response patterns^19^.

In this study, respiratory rate decreased, respiratory cycle duration, inspiratory time and tidal volume increased during two-minute ELF-EMF exposure at 50 Hz frequency and 0.3 mT magnetic flux density. These findings indicate that EMF exposure may cause some changes in respiratory functions. The limitation of this study can be considered as short-term and single-intensity ELF-EMF exposure. On the other hand, it is a reference for future studies to investigate the potential effects of EMF exposure on respiratory functions under long-term and different-intensity exposure conditions.

## CONCLUSION

A decrease in respiratory rate and an increase in respiratory cycle duration, inspiratory time, and tidal volume were observed with short-term ELF-EMF exposure of 2 min. Furthermore, the changes in these parameters disappeared 2 min after the end of exposure.
